# Systemic Immune Factors and Risk of Allergic Contact Dermatitis: A Bidirectional Mendelian Randomization Study

**DOI:** 10.3390/ijms251910436

**Published:** 2024-09-27

**Authors:** Yingxin Long, Wenzhang Dai, Kexin Cai, Yuan Xiao, Anqi Luo, Ziwei Lai, Junlin Wang, Lipeng Xu, Hong Nie

**Affiliations:** State Key Laboratory of Bioactive Molecules and Druggability Assessment, Guangdong Province Key Laboratory of Pharmacodynamic Constituents of TCM and New Drugs Research, International Cooperative Laboratory of Traditional Chinese Medicine Modernization and Innovative Drug Development of Chinese Ministry of Education (MOE), College of Pharmacy, Jinan University, 601 Huangpu Avenue West, Guangzhou 510632, China; mabu66@163.com (Y.L.); daiwen6811@outlook.com (W.D.); caikexin0000@163.com (K.C.); yuanhsiao@163.com (Y.X.); luo19991223@126.com (A.L.); laiziwei2004@163.com (Z.L.); wangjunlin@jnu.edu.cn (J.W.)

**Keywords:** cytokines, immune factors, allergic contact dermatitis, Mendelian randomization

## Abstract

Skin inflammation and immune regulation have been suggested to be associated with allergic contact dermatitis (ACD) progression, but whether the system’s immune regulation is a cause or a potential mechanism is still unknown. This study aims to assess the upstream and downstream of systemic immune factors on ACD within a bidirectional Mendelian-randomization design. A bidirectional two-sample MR analysis was employed to implement the results from genome-wide association studies for 52 system immune factors and ACD. Genetic associations with systemic immune factors and ACD were obtained from the IEU Open GWAS project database. The inverse-variance weighted (IVW) method was adopted as the primary MR analysis, MR-Egger, weighted median, MR-pleiotropy residual sum, and outlier (MR-PRESSO) was also used as the sensitivity analyses. Only Tumor necrosis factor ligand superfamily member 11 (TNFS11) from among 52 systemic immune factors was associated with a protective effect of ACD. However, ACD was associated with a decrease in Interleukin-9 (IL9) and an increase in C-X-C motif chemokine 1 (GROα), Tumor necrosis factor ligand superfamily member 10 (TRAIL), C4, and complement factor B of the assessed systemic immune factors. This study identified TNFS11 as the upstream regulator and IL9, GROα, TRAIL, C4, and complement factor B as the downstream regulator of ACD, providing opportunities for new therapeutic exploitation of ACD. Nonetheless, these associations of systemic immune factors need to be verified in vivo.

## 1. Introduction

Immune dysregulation and inflammation under uncheck may result in autoimmune, autoinflammatory disorders, neurodegenerative disease, skin hypersensitivity, or even cancer [1]. Allergic contact dermatitis (ACD) is an inflammatory skin disease accompanied by itch and pain. It occurs in up to 1 in 5 people; anyone can be affected at any age [2], leading to considerable impairment in quality of life and economic losses. Allergens come from daily use in occupational or normal life, including ingredients of cosmetics, chemicals, and medicaments [3]. ACD is a type IV delayed hypersensitivity reaction mediated by specific T cells. Once the organism is exposed to an allergen for the first time, the hapten is incorporated by dendritic cells and transported to the skin-draining lymph nodes. When exposure to the same allergen again, it triggers invasion of these T cells and results in ACD [4,5]. In this progress, keratinocytes expressed proinflammatory cytokines (IL1α, IL1β, TNF-α, and IL6) and chemokines (IP-10, MCP-1, RANTES, and CCL18), epidermal Langerhans express MHC class Ⅰ and Ⅱ molecules, and migrate to the skin-drain lymph nodes [6]. Skin inflammation and immune regulation are closely associated with ACD progression, but whether the immune system factor is a cause or a potential mechanism is still unknown. In addition, the potential etiologies may include oxidative stress, whereby a compromised antioxidant response to allergen-induced oxidative stress can result in allergic sensitization [7].

Mendelian randomization (MR) is a research method that uses genetic variation in non-experimental data to infer a causal relationship between exposure and outcome variables [8]. The principle of MR refers to Mendel’s second law of the independent separation of genetic alleles when DNA is passed from parent to offspring during gametic formation. It can reveal potential relationships between risk factors and diseases without requiring large sample sizes and long-term follow-up to obtain results [9]. Bidirectional MR, as an extension of traditional MR, can reveal whether there is a feedback loop between exposure and outcome variables [10]. Chris [11] demonstrated that specific systemic inflammatory regulators may be downstream effects of Alzheimer’s disease. Zheng [12] found that eight cardiometabolic risk factors showed causal effects on CKD in Europeans. ACD is closely associated with immune response; however, studies of the Mendelian randomization method to explore the causal effect between immune factors and ACD are still scarce. Therefore, we used two-sample two-way MR to identify upstream regulators and downstream effectors of ACD.

This study aims to explore the association between systemic immune factors and ACD within a bidirectional MR design using up-to-date genome-wide association summary-level data to assess the causal association and the direction of association between systemic immune factors and ACD.

## 2. Results

### 2.1. Genetically Predicted Systemic Immune Factors on Risk of Allergic Contact Dermatitis

**Figure 1:**
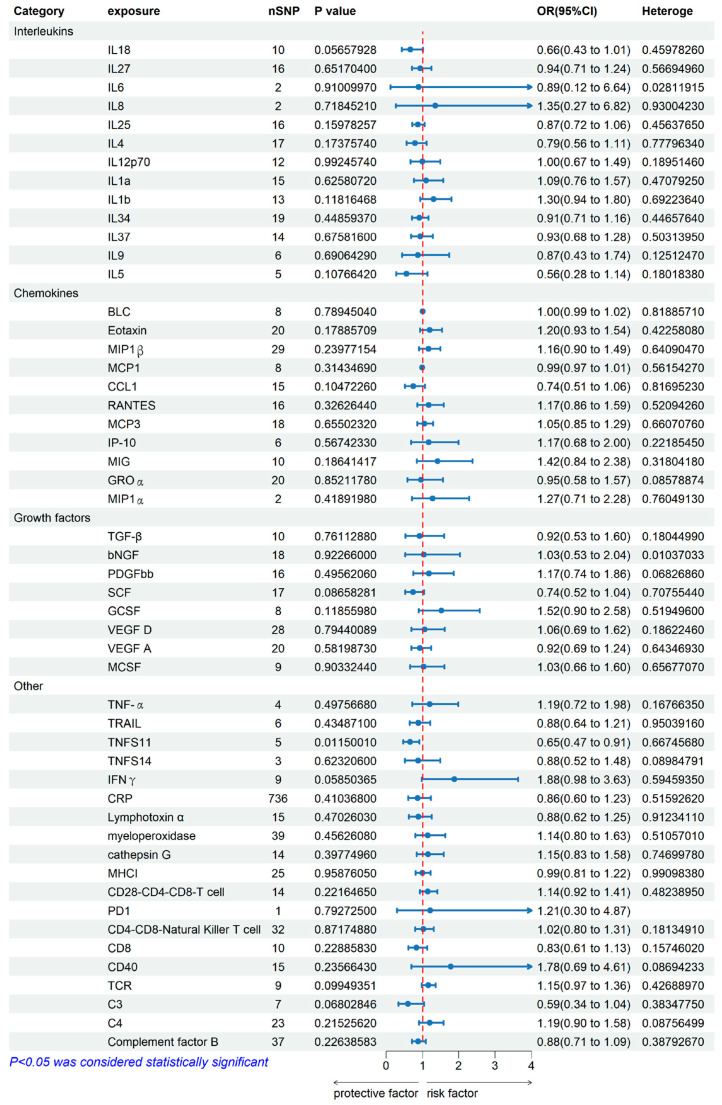
Association of systemic immune factors (Interleukin, Chemokines, Growth factors, and Others) with ACD using Mendelian randomization. The odds ratio (OR) and 95% confidence interval (CI) represent the change in the odds ratio of ACD per 1-SD increase in the systemic immune factors level. IL18, Interleukin-18; IL27, Interleukin-27; IL6, Interleukin-6; IL8, Interleukin-8; IL25, Interleukin-25, IL4,Interleukin-4; IL12p70, Interleukin-12p7; IL1α, Interleukin-1α, IL1β, Interleukin-1β; IL34, Interleukin-34; IL37, Interleukin-37; IL9, Interleukin-9; IL5, Interleukin-5; BLC, C-X-C Motif Chemokine Ligand 13; MIP1b, C-C Motif Chemokine Ligand 4; MCP1, C-C Motif Chemokine Ligand 2; CCL1, C-C Motif Chemokine Ligand 1; RANTES, C-C Motif Chemokine Ligand 5; MCP3, C-C Motif Chemokine Ligand 7; IP-10, C-X-C motif chemokine 10; MIG, C-X-C motif chemokine 9; GROα, C-X-C motif chemokine 1; MIP1α, C-C motif chemokine; TGF-β, Transforming Growth Factor Beta; bNGF, beta-nerve growth factor; PDGFbb, Platelet Derived Growth Factor Subunit B; SCF, Klotho; GCSF, Granulocyte colony-stimulating factor; VEGF D, vascular endothelial growth factor D; VEGF A, vascular endothelial growth factor A; MCSF, Macrophage colony-stimulating factor; TNF-α, Tumor Necrosis Factor alpha; TRAIL, Tumor necrosis factor ligand superfamily member 10; TNFS11, Tumor necrosis factor ligand superfamily member 11; TNFS14, Tumor necrosis factor ligand superfamily member 14; IFNγ, Interferon gamma; CRP, c-reactive protein; Lymphotoxin α, Lymphotoxin Alpha; MHCI, MHC class I polypeptide.

**Figure 2:**
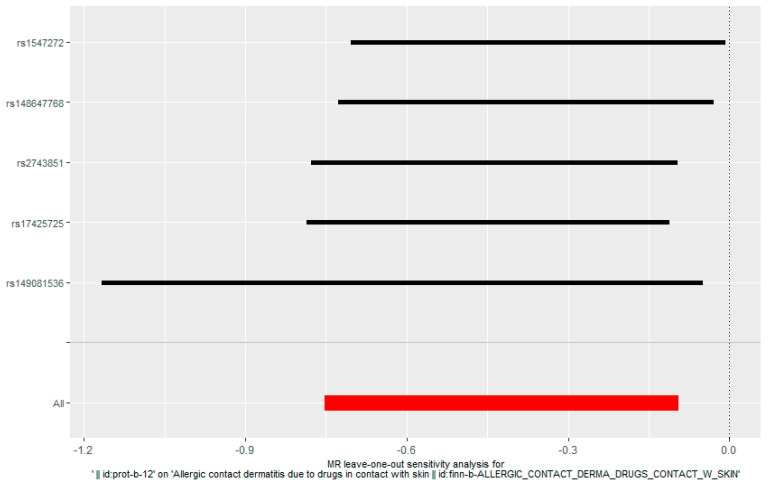
Causal relationship between RANKL and ACD in the results of the ‘leave-one-out’ analysis in the forward analysis, the red points showed the combined causal estimate using all SNPs together in a single instrument.

When using systemic immune factors as the exposure, Figure 1 and Figure 2 showed the main results from the two-sample MR study of systemic immune factors on the risk of allergic contact dermatitis. Among the 52 available systemic immune factors, only IL6, IL8, MIP1a, and PD1 had three or fewer independent genome-wide significant SNPs when using the cut-off (*p* < 5 × 10^−6^). F-statistics were more than 10, suggesting that weak instrument bias may not be substantial. Details of the SNPs included are in Supplementary Tables S2 and S3. As the Supplementary Tables S4 and S5, Figure 1 and Figure 2 shown, only Tumor necrosis factor ligand superfamily member 11 (TNFS11) among 52 systemic immune factors showed the inverse association with ACD using IVW analysis. The combined ORs of ACD were 0.65 [95% CI: 0.47, 0.91] per a 1-SD increase in SCGFb. Subsequently, the multiple comparisons were corrected using IVW with inconsistent estimates from sensitivity analyses.

In the category of interleukins and growth factors. MR-Egger intercept detection showed that vascular endothelial growth factor D (*p*-value < 0.05) is potential horizontal pleiotropy. IVW heterogeneity tests showed that heterogeneity of Interleukin-6 and beta-nerve growth factor are 0.028119, 0.01037033 (*p*-value > 0.05), it elucidated that Interleukin-6 and beta-nerve growth factor were heterogeneity. Leave-one-out studies were used for sensitivity analysis, and it showed no driver SNP.

The IVW estimates a potential effect of TNFS11 may decrease the risk of ACD. The bidirectional MR estimates using five SNPs as instrumental variables are presented in Figure 2. This forest map illustrates SNPs, which are mutations or variations occurring at a specific gene position in the DNA sequence. Each level represents a distinct SNP, with multiple SNPs influencing ACD. All results were statistically significant (*p* < 0.05) and consistently indicated a causal effect of TNFS11 and ACD.

### 2.2. Genetically Predicted Allergic Contact Dermatitis on Systemic Immune Factor Levels

**Figure 3:**
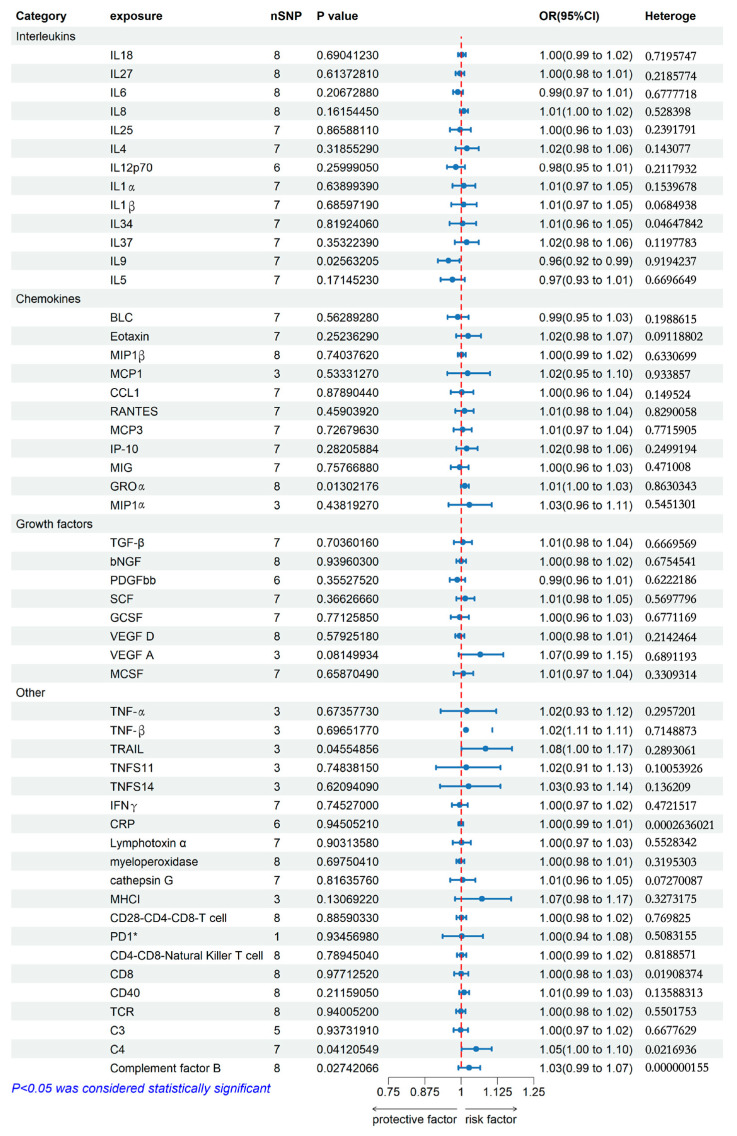
Association of ACD with systemic immune factors (Interleukin and Chemokines) using Mendelian randomization. The beta and 95% confidence interval (CI) represent the change in the SD of system immune factors per log odds increase in ACD. IL18, Interleukin-18; IL27, Interleukin-27; IL6, Interleukin-6; IL8, Interleukin-8; IL25, Interleukin-25; IL4, Interleukin-4; IL12p70, Interleukin-12p7; IL1α, Interleukin-1α, IL1β, Interleukin-1β; IL34, Interleukin-34; IL37, Interleukin-37; IL9, Interleukin-9; IL5, Interleukin-5; BLC, C-X-C Motif Chemokine Ligand 13; MIP1b, C-C Motif Chemokine Ligand 4; MCP1, C-C Motif Chemokine Ligand 2; CCL1, C-C Motif Chemokine Ligand 1; RANTES, C-C Motif Chemokine Ligand 5; MCP3, C-C Motif Chemokine Ligand 7; IP-10, C-X-C motif chemokine 10; MIG, C-X-C motif chemokine 9; GROα, C-X-C motif chemokine 1; MIP1α, C-C motif chemokine 3; TGF-β, Transforming Growth Factor Beta; bNGF, beta-nerve growth factor; PDGFbb, Platelet Derived Growth Factor Subunit B; SCF, Klotho; GCSF, Granulocyte colony-stimulating factor; VEGF D, vascular endothelial growth factor D; VEGF A, vascular endothelial growth factor A; MCSF, Macrophage colony-stimulating factor; TNF-α, Tumor Necrosis Factor alpha; TNF-β, Tumor Necrosis Factor beta; TRAIL, Tumor necrosis factor ligand superfamily member 10; TNFS11, Tumor necrosis factor ligand superfamily member 11; TNFS14, Tumor necrosis factor ligand superfamily member 14; IFNγ, Interferon gamma; CRP, c-reactive protein; Lymphotoxin α, Lymphotoxin Alpha; MHCI, MHC class I polypeptide, * means SNP less than 3.

**Figure 4:**
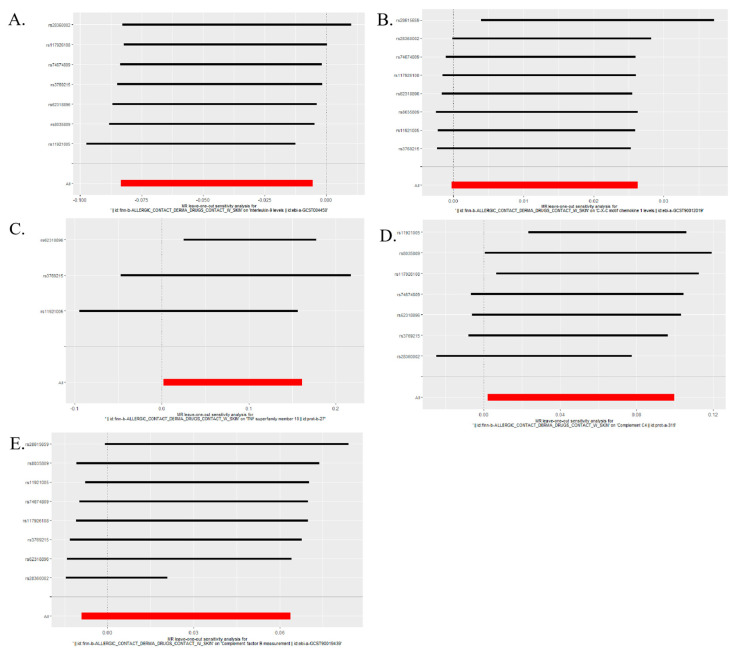
Causal relationship between ACD and 5 immune factors—IL9 (**A**), GROα (**B**), TRAIL (**C**), C4 (**D**), and complement factor B (**E**) in the results of ‘leave-one-out’ analysis in the forward analysis, the red points showed the combined causal estimate using all SNPs together in a single instrument.

When using ACD as the exposure, Figure 3 and Figure 4 showed that ACD had a suggestive positive association with decreased IL9 in the interleukin family using IVW analysis. ACD was associated with a lower risk of IL9 [odds ratio (OR): 0.96, 95% confidence interval (CI): 0.92–0.99].

In the category of Chemokines, a suggestive inverse association was also found that ACD had a suggestive positive association with increased C-X-C motif chemokine 1 (GROα), and ACD was associated with a higher risk [OR: 1.01, 95% CI: 1.00–1.03] for GROα. MR-Egger intercept detection showed that IL9 (*p*-value < 0.05) was potential horizontal pleiotropy. Moreover, the IVW heterogeneity tests showed that interleukin-34 (IL34) was heterogeneous (*p*-value > 0.05).

In the category of others, the suggestive association showed that ACD had a suggestive positive association with increased Tumor necrosis factor ligand superfamily member 10 (TRAIL), C4, and Complement factor B using IVW analysis. ACD was associated with a higher risk [OR: 1.08, 95% CI: 1.00–1.17 for TRAIL, OR: 1.05, 95% CI: 1.00–1.10 for C4, and OR: 1.03, 95% CI: 0.99–1.07 for Complement factor B]. MR-Egger intercept detection showed that CD4-CD8-Natural Killer T cell, CD8, CD40, TCR, C3, C4, and Complement factor B (*p*-value < 0.05) were potential horizontal pleiotropy. In comparison, IVW heterogeneity tests showed that interleukin-34 (IL34), c-reactive protein (CRP), CD8, and C4 were heterogeneous (*p*-value > 0.05). Leave-one-out studies were used for sensitivity analysis and it also showed that it has no driver SNP.

The IVW estimate suggested that the ACD may result in a decrease in IL9 and an increase in GROα, TRAIL, C4, and complement factor B. The bidirectional MR estimates are shown in Figure 4. Using the seven SNPs of ACD on IL9 (Figure 4A), eight SNPs of ACD on GROα (Figure 4B), three SNPs of ACD on TRAIL (Figure 4C), seven SNPs of ACD on C4 (Figure 4D), and eight SNPs of ACD on complement factor B (Figure 4E).

## 3. Discussion

In this study, bidirectional MR analysis was performed using the latest genome-wide association summary level data to assess the causal relationship between systemic immune factors and ACD. We used a bidirectional analysis strategy to distinguish between upstream and downstream in the disease and expound reverse causality. To ensure reliability and reduce the pleiotropic effect of MR, we incorporated multiple MR approaches, such as MR-PRESSO and MR-Egger.

In this bidirectional MR analysis, we found that TNFS11 was associated with a reduced risk of ACD. At the same time, the results also indicated that ACD was associated with a decreased level of IL9 and increased levels of GROα, TRAIL, C4, and complement factor B levels, suggesting there is a pro-inflammatory response in the ACD.

The pathogenesis of ACD is complex. The currently used clinical topical corticosteroids suppress redness and scratching symptoms associated with ACD, but have no distinctive effect to address the underlying immune dysfunction [13]. There is a lack of effective medicine for the treatment of ACD, so it is important to explore its mechanism.

ACD is mediated by many cell types within the immune system; some systemic immune factors are regulated by these cells to act. For example, dendritic cells, TH1 and TH17 cells release inflammatory cytokines, regulatory T cells (TREGs) play a role in inhibiting inflammation, and Th17/Treg imbalance is involved in several autoimmune, inflammatory and allergic reactions in ACD patients [14]. Effector T lymphocytes and memory T lymphocytes are key adaptive immune mediators of ACD, and natural killer (NK) and innate lymphocytes are also believed to contribute to the occurrence and development of ACD [15]. Our findings validate the hypothesis that some systemic immune factors play an important role in the progression of ACD.

TNFS11 is a receptor activator of NF-κB ligand, well known as RANKL, and interacts with T cells and dendritic cells in osteoclasts [16]. A previous study showed that the increased TNFS11 in serum may be involved in the pathogenesis of skin dermatitis via dendritic cells, T cells, and keratinocytes in inflamed skin or systemic inflammatory conditions [17]. The expression of TNFS11 has been demonstrated in keratinocytes, and activation of RANKL induces functional changes in dendritic cells (DC) along with altered expression of DC surface receptors associated with immunosuppressive function [18]. RANKL confers resistance to apoptosis in Langerhans cells (LC) and interacts with RANK on LC, thereby promoting the expansion of regulatory T cells. Additionally, TNF-α produced by RANKL-stimulated DC is recognized as a crucial mediator of regulatory T-cell expansion [19]. Immunosuppression is used to treat many diseases in clinical practice, such as psoriasis [19,20]. However, studies on the association between TNFS11 and ACD are still scarce.

IL9 is a gamma chain receptor family cytokine that uses the common IL-2 receptor gamma chain for signal transduction [21]. It has a variety of sources, including TH2 cells and TH9 cells, which are involved in the pathogenesis of ACD, atopic dermatitis, contact dermatitis, and other skin allergies [22]. IL9 is often associated with early atopic sensitization [23] and is a key mediator of regulatory T cells in immunosuppression [22]. On the other hand, previous research suggests that IL9 produced by TH9 cells is temporary, peaking in circulating T9 cells the day after stimulation. Temporary production and subsequent down-regulation of IL9 was also observed in TH9 cells differentiated in vitro [24,25].

Therefore, it can be explained that the expression of IL9 predicted by Mendelian randomization is inconsistent with the remaining several validators, such as GROα. TRAIL, offering potential explanations—such as statistical power or biological variability—would help contextualize those findings.

GROα, also named C-X-C motif chemokine ligand 1, is a chemotactic agent of neutrophils and has been implicated in a variety of inflammatory-induced diseases [26]. Stimulated keratinocytes in psoriatic lesions release a variety of cytokines, such as GROα that have a chemotactic effect on neutrophils, which accumulate under the stratum corneum, a highly inflammatory area of psoriatic lesions. They are attracted to and activated by the synergistic action of chemokines such as GROα, creating a vicious cycle of inflammation maintenance [27,28]. Tumor necrosis factor-associated apoptosis-inducing ligand (TRAIL) can induce apoptosis of cancer cells without causing toxicity in mice [29]. TRAIL drives cell death and dermatitis, inhibition of TRAIL-induced cell death can prevent fatal dermatitis, and TNF inhibition combined with TRAIL inhibition improves the development of dermatitis in autoimmune patients [30]. Complement C4, one of the main components of innate immunity that immediately recognizes and eliminates invading microorganisms, is a key molecule in the complement system [31]. The role of C4 cleavage and C4a production as activating products in trauma significantly increased serum C4 levels in patients with atopic dermatitis and psoriasis [32]; a decrease in serum C4 levels represents an increase in anti-inflammatory effects [33]. Complement C4 may be associated with a proinflammatory state at the affected site, accompanied by tissue edema and hypoxia, leading to organ dysfunction [34]. Complement factor B (CBF), a serine protease, acts as a C3 convertase to activate the complement bypass pathway [35], and its mediated activation of the complement bypass pathway can cause inflammation [36]. Macrophages are an important site of CBF synthesis and may significantly contribute to the local concentration of CBF at the inflammatory site. Inflammatory cytokines IFN-γ and TNF-α at the inflammatory site induce the activation of CBF, thus further aggravating the development of inflammation [35].

We acknowledge that this study has some limitations. First, all data involved in the analysis were collected from individuals of European descent, which limits the general applicability of our findings to other ethnic groups. It may lead to biased estimates and affect generalizations. The generalization of the findings to other populations should be approached with caution, and further research, such as in vivo or in vitro studies or studies involving more diverse cohorts, may be necessary to address any concerns regarding the applicability. Second, MR analyses often reveal lifetime exposures, so the size of the impact of exposure may be overestimated, and the impact of exposure should be further examined in vivo and in vitro experiments. Third, ineffective findings for other immune factors may reflect lower statistical efficacy. The lack of statistical power may explain the inconsistencies in results between systemic and diseased sites and should be further validated [37,38].

Furthermore, elucidating the potential clinical applications of the role of TNFS11 would enhance the practical impact of this study. Previous studies have demonstrated the immunosuppressive effect of TNFS11, while the clinical treatment of immunosuppressive methods for allergic skin diseases is widely used, such as vitamin C, ultraviolet irradiation, and immunosuppressive agents. However, the role of TNSF11 in ACD treatment has not been fully recognized. Therefore, our study establishing a causal relationship between TNFS11 and ACD highlights the importance of considering TNFS11-activated medicine as a potential treatment method for improving quality of life and alleviating symptoms associated with ACD.

In the future, we will validate studies in vivo and in vitro. Our research group has reported much research about SADBE-induced ACD; we will further validate whether RANKL protects humans from ACD, or ACD downregulates IL9 and upregulates the expression of GROα, TRAIL, C4, and Complement factor B.

## 4. Methods

### 4.1. MR and Assumptions

We used a bidirectional two-sample Mendelian randomization (MR) study by using genetic instruments—single-nucleotide polymorphisms (SNPs)—to predict systemic immune factors and ACD from the latest GWAS [11]. Bidirectional was designed to assess the association of systemic immune factors with ACD as well as to test whether ACD causes systemic inflammatory regulators. MR studies rely on three fundamental assumptions: the genetic variants exposure is strongly associated with the exposure of interest; the genetic variants exposure is independent of unmeasured confounders of the exposure–outcome relation; the genetic variant exposure influences the outcome only through the exposure of interest [39].

### 4.2. Genetic Associations with Systemic Immune Factors and Allergic Contact Dermatitis

Genetic variants exposure of cytokines, Interleukins, chemokines, and other systemic immune factors were obtained from the IEU OpenGWAS project database (https://gwas.mrcieu.ac.uk/) of 52 systemic immune factors (accessed on 25 May 2024). GWAS ID of systemic immune factors in this study are listed in Supplementary Table S1. Genetic variants exposure to ACD were also obtained from the IEU OpenGWAS project database (https://gwas.mrcieu.ac.uk/) (accessed on 25 May 2024). We selected the trait with “Allergic contact dermatitis due to drugs in contact with skin”, with the GWAS ID “finn-b-ALLERGIC_CONTACT_DERMA_DRUGS_CONTACT_W_SKIN”. Disease GWAS included 296 European patients with ACD due to drugs in contact with the skin and 218,496 European controls. We obtained the number of SNPs of 16,380,466 from this GWAS.

### 4.3. Selection of Genetic Instruments

Since SNP–depression association reached the genome-wide significant threshold [*p* < 5 × 10^−8^] is scarce, we choose the suggested significance level [*p* < 1 × 10^−6^] to extract instrumental variables; this study aims to disentangle bidirectional relationships between systemic immune factors and ACD in MR analyses. Subsequently, we performed the clumping procedure selected by linkage disequilibrium clumping [r^2^ > 0.01] and a window size = 10,000 kb with the European ancestral individuals from the 1000 Genomes Project to exclude SNPs that were in strong linkage disequilibrium.

### 4.4. Statistical Analysis

**Figure 5:**
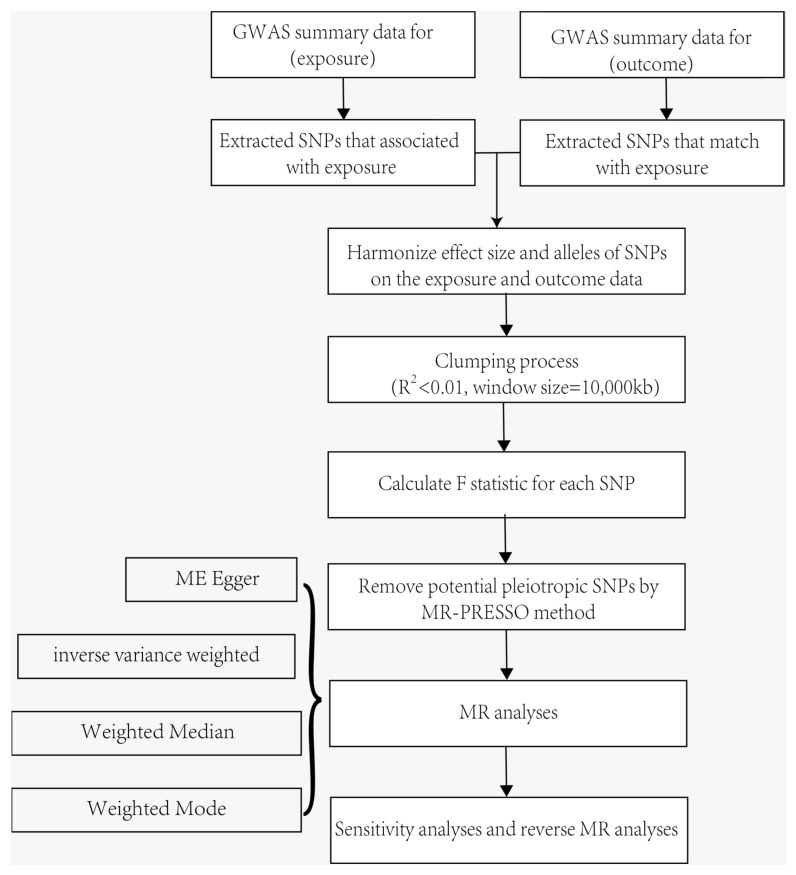
Datasets, assumptions, and study design of the bidirectional Mendelian randomization study of the associations between 52 immune factors and ACD.

In this study, we applied multiple approaches, including the inverse variance weighted (IVW), the MR-Egger regression, and the weighted median, to estimate the causal effects of exposures on outcomes [40]. We selected IVW as the major analysis method to estimate the potential bidirectional causal associations between system immune factors and ACD. The MR-Egger regression and the weighted median were for alternative analyses. We used Q statistic, MR Egger intercept, MR-PRESSO global test, and knock-out test to test the heterogeneity, pleiotropy, and driver SNPs. A brief description of the bidirectional MR design is displayed in Figure 5.

## 5. Conclusions

As for the association between ACD and systematic immune factors, we found that only TNFS11 showed a suggestive positive association with ACD. Our findings suggest that systemic immune responses and systemic inflammation may not cause ACD but rather are downstream consequences of ACD.

## Data Availability

The original contributions presented in the study are included in the article/Supplementary Materials, further inquiries can be directed to the corresponding author/s.

## Supplementary Materials

The supporting information can be downloaded at: https://www.mdpi.com/article/10.3390/ijms251910436/s1.

## Abbreviations

CI, confidence interval; GWASs, genome-wide association studies; IVW, inverse-variance weighted; MR, Mendelian randomization; MR-PRESSO, MR-pleiotropy residual sum and outliers; OR, odds ratio; SNPs, single nucleotide polymorphisms.
